# Unprovoked venous thromboembolism recurrence and arterial embolism revealing lung cancer: a case report

**DOI:** 10.1186/s12959-024-00622-7

**Published:** 2024-06-18

**Authors:** Maria-Cristina Glodeanu, Victoria Mutruc, Camelia-Maria Apetrei, Manuela Ursaru, Laurentiu Sorodoc, Catalina Lionte

**Affiliations:** 1 2nd Internal Medicine Clinic, “Sf. Spiridon” Emergency Clinical County Hospital, Iasi, Romania; 2 Rheumatology Department, Clinical Recovery Hospital, Iasi, Romania; 3 Radiology Department, “Sf. Spiridon” Emergency Clinical County Hospital, “Grigore T. Popa” University of Medicine and Pharmacy, Iasi, Romania; 4 Internal Medicine Department, “Grigore T. Popa” University of Medicine and Pharmacy, Iasi, Romania

**Keywords:** Venous thromboembolism, Arterial embolism, Occult cancer, Screening

## Abstract

The link between venous thromboembolism (VTE) and cancer is well known. VTE could be the initial sign of an occult malignancy. There are more diagnoses of cancer after an unprovoked VTE compared to a provoked VTE, with a reported prevalence between 4.5% and 5.6% over 12 months, within the first 6 months of VTE diagnosis. There are no recommended guidelines and scores yet adopted in clinical practice, but many studies support occult cancer screening in unprovoked VTE patients. We report the case of a patient with a history of unprovoked pulmonary embolism (PE) diagnosed with bronchopulmonary neoplasm in an advanced stage one year after the thromboembolic event. When the cancer was first diagnosed, the patient’s condition was already serious, being too late for the adoption of measures meant to decrease the risk of mortality and increase the duration of survival. We wanted to emphasize the importance of occult cancer screening in patients with unprovoked VTE and the fact that early cancer diagnosis reduces the risk of cancer progression, decreasing mortality and morbidity related to it.

## Background

The venous thromboembolism (VTE), malignancy association initially emphasized by Armand Trousseau in the early 19th century constitutes a significant cause of morbidity and mortality to this day [1]. VTE can be the first sign of cancer and can precede the oncological diagnosis by up to 6 years [2]. Recent studies have detected a much lower interval between the VTE and cancer diagnosis in these patients. The SOME study, conducted in Canada [3] and the MVTEP, conducted in France [4] estimated that 4.5% and respectively 5.6% of patients with unprovoked VTE were diagnosed with occult cancer in the following 12 months, most cancers being diagnosed in the first 6 months [3]. Other studies suggest that 7.6% of patients with unprovoked VTE events develop cancer during follow-up, compared to only 1.9% of patients with provoked VTE [5]. Data extracted from the California Cancer Registry revealed that 596 (0.11%) of 528,693 cancer cases were related to a previous diagnosis of unprovoked VTE within 1 year of cancer diagnosis [1]. Patients with unprovoked VTE during occult cancer have a higher risk of VTE recurrence compared to patients with clinically diagnosed cancer. The risk of VTE recurrence is associated with the presence of more prothrombotic cancers, diagnosed in advanced stages, including lung cancer [6]. The California Cancer Registry developed a large study where among 91,933 patients newly diagnosed with lung cancer in the first and second year post-unprovoked VTE, the incidence rate was 3% in patients with non-small cell lung cancer (NSCLC) and 3.4% among patients with small cell lung cancer (SCLC) [7].

## Case presentation

**Fig. 1:**
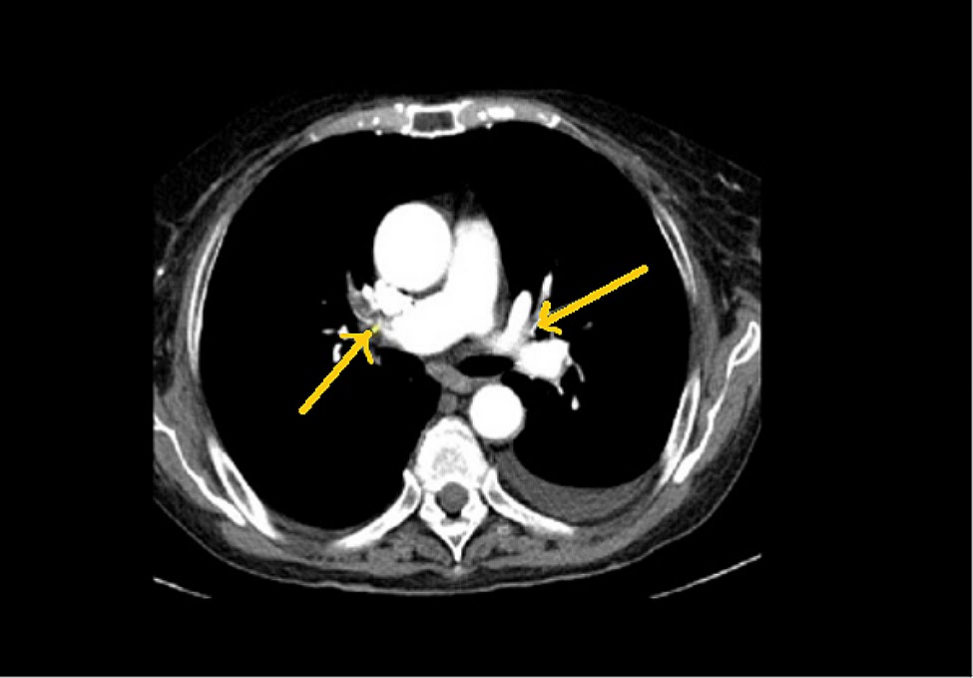
Initial bilateral pulmonary thrombembolism. Angio-CT examination, axial section, reveals thrombus in pulmonary artery bilateral (yellow arrows)

A 56-year-old male patient, former smoker (20 packs per year), exposed to occupational pollutants (worker in the metallurgical industry, exposed to metal vapours which may be generated during melting and welding for 20 years), was admitted to the Internal Medicine Department with dyspnea on mild exertion. According to the personal medical history, the patient is known to have hereditary spherocytosis, having undergone a splenectomy at the age of 5, as well as episode of unprovoked pulmonary thromboembolism (PE) 11 months prior to admission in our department, confirmed by pulmonary angio-CT examination (Fig. 1), receiving since an oral anticoagulant treatment (OAC) with rivaroxaban 20 mg/day. He also suffers from essential arterial hypertension grade 3 with a very high cardiovascular risk (treated with a fixed dose combination of perindopril 10 mg, indapamide 2.5 mg, amlodipine 5 mg o.d.), chronic obstructive pulmonary disease (COPD) GOLD stage II (treated with inhaled indacaterol 150 mcg o.d.). The patient is known to have had multiple emergency presentations in a local emergency hospital for episodes of significant dyspnea, with another pulmonary angio-CT examination performed three months before being admitted to our department, which revealed a partial filling defect in the right subsegmental pulmonary artery and multiple mediastinal adenopathy. We note that until the first episode of PE, the patient had not experienced dyspnea and had no risk factors for provoked VTE (i.e. surgery, acute illness, confine to bed more than 3 days, leg injury, inflammatory or autoimmune diseases, etc.). Upon admission, the physical examination revealed a hemodynamically stable patient, with oxygen saturation in arterial blood between 90 − 85% room air, no palpable superficial ganglia, no chronic venous changes in the lower limbs.

Blood tests revealed polyglobulia (Hb 18.9 mg/dL, hematocrit 55.5%), mild thrombocytopenia (128,000/µL), and peripheral blood smear showed erythrocyte anisocytosis, rare platelets (macro thrombocytes and megalothrombocytes), elevated CA 125 (646 U/mL; the normal value < 35 U/ml), neuron-specific enolase (NSE) slightly elevated (18.4 ng/mL; the normal value < 16,3 ng/mL), the other tumor markers (AFP, CA19-9, ACE) being within normal limits. Upon hospitalization, we did not performed thrombophilia testing since the patient was already on anticoagulant therapy, which can influence the results. A transthoracic echocardiography (TTE) was performed, which revealed right ventricle dysfunction (TAPSE 16 mm), probability of secondary pulmonary hypertension (PH) with an estimated systolic pressure in pulmonary artery of 41 mmHg, hypertensive cardiopathy with diastolic dysfunction and a mildly reduced left ventricular ejection fraction (46%). During the hospitalization, the patient presented sensitivity disorders on the right hemibody, lasting 2–3 min, with complete recovery. The neurologist diagnosed him with transient ischemic attack. Up to this point, we concluded that secondary PH could be interpreted in the context of PE superimposed on an underlying pulmonary lesion, and polyglobulia being secondary, physiologically appropriate to chronic hypoxia. Considering the increased values of tumor markers CA 125 and NSE, the existence of adenopathy determined by previous CT examinations, the suspicion of neoplasia was raised. The patient was scheduled for a thoraco-abdominal-pelvic CT examination with contrast material within 6 weeks. During hopitalization, the patient was treated with low-molecular weight heparin - LMWH (enoxaparin 1 mg/body weight), antihypertensives (fixed dose combination perindopril 10 mg, indapamide 2.5 mg, and amlodipine 10 mg, associated with nebivolol 5 mg, rilmenidine 1 mg), statins (fixed dose combination rosuvastatin 10 mg, ezetimibe 10 mg), heart failure medication (spironolactone 25 mg, empagliflozin 10 mg), and he continued the COPD treatment. The same COPD, hypertension and heart failure medication was prescribed upon discharge, and we recommended OAC therapy with apixaban 5 mg b.i.d.

**Fig. 2:**
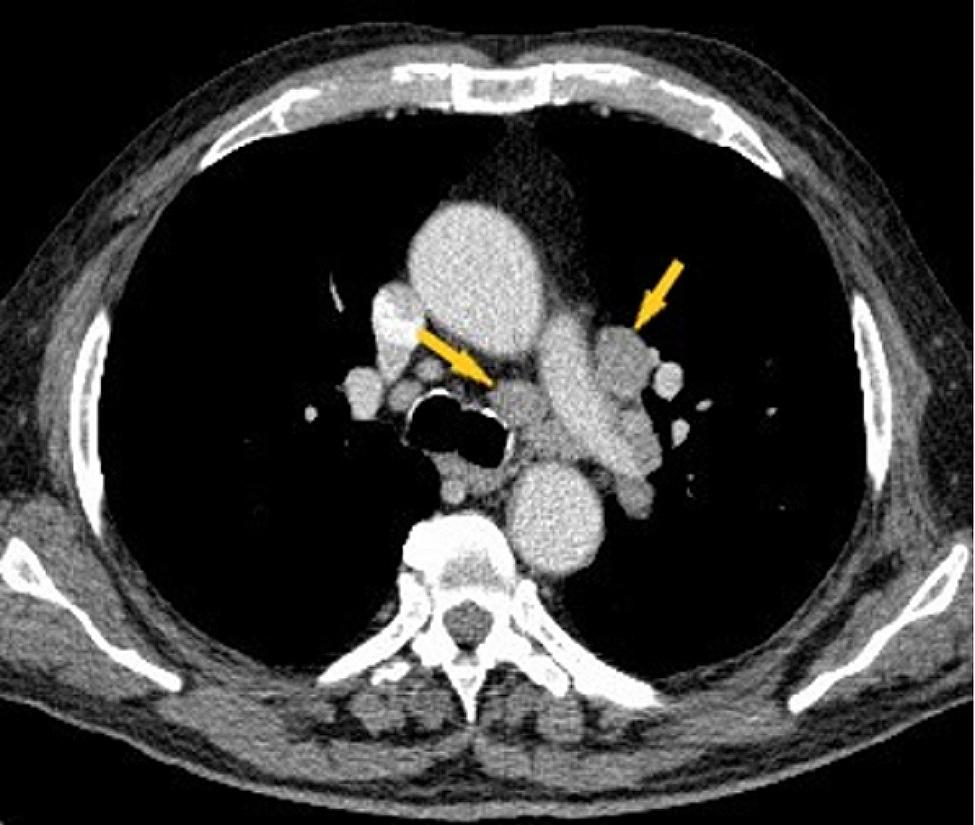
Chest CT examination, venous time, axial section, mediastinal window: mediastinal adenopathy involving the left branch of the pulmonary artery (yellow arrows)

**Fig. 3:**
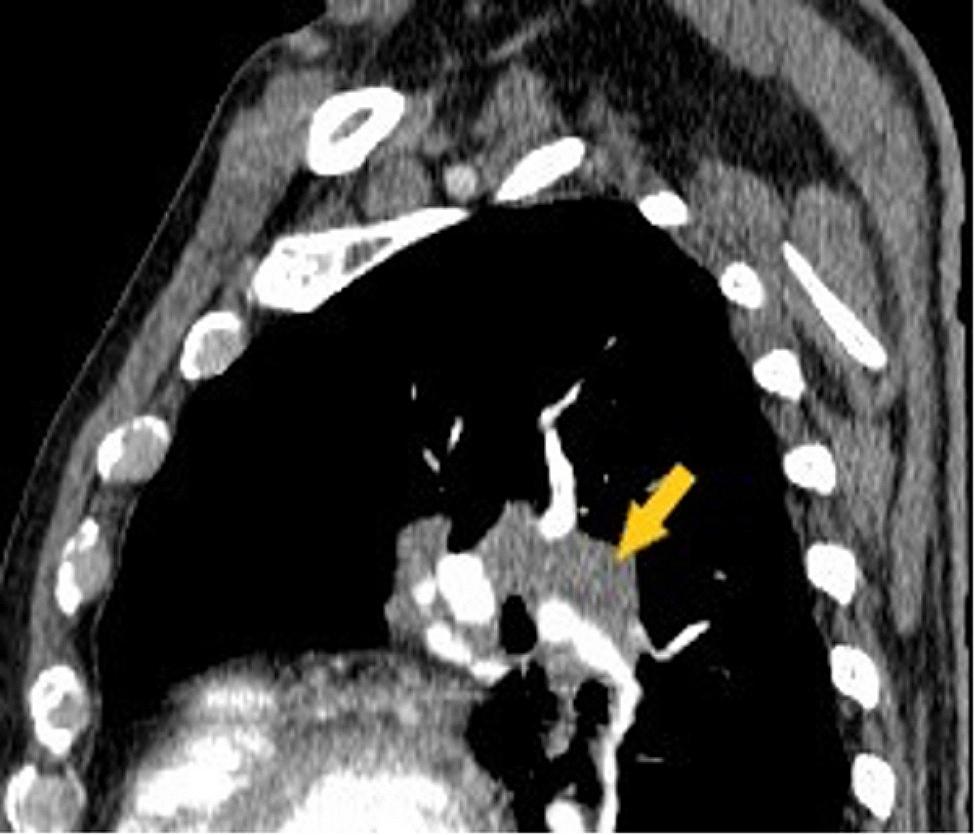
Chest CT examination, arterial time: lesional complex left hilum tumor mass-adenopathy (yellow arrow)

**Fig. 4:**
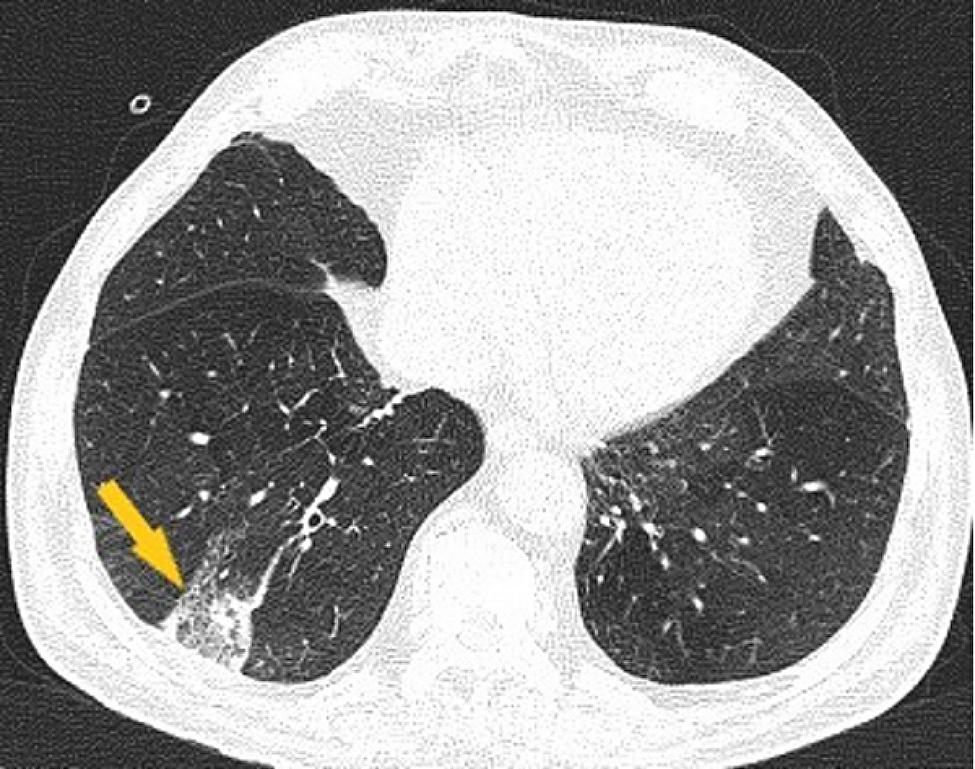
Chest CT examination, arterial time: right lower lobe with subpleural consolidation suggestive of pulmonary infarction (yellow arrow)

One month after leaving our department, the patient’s general condition worsens with respiratory failure manifested by aggravating dyspnea, oxygen saturation in the arterial blood 70% room air, being admitted to a secondary hospital in the territory. The unfavorable evolution with the persistence of severe respiratory insufficiency required the transfer of the patient to the Pneumology Department of a tertiary hospital, where fibrobronchoscopy with biopsy was performed, the microscopic result excluding the existence of tumor aspects in the examined section. Afterwards, a thoraco-abdominal-pelvic CT was performed, which highlights bilateral mediastino-hylar adenopathy with homogeneous contrast uptake with very varied sizes, asymmetric, more important on the left side. The left hilar adenopathies are more voluminous, covering the left branch of the pulmonary artery, the left primitive and the left upper lobar bronchus. A lesional complex made up of a tumor mass and adenopathy without a demarcation plan (Figs. 2 and 3) was also possible; inadequate opacification at the level of the right basal sub-segmental arteries (Fig. 4) suggesting lung infarction. The patient’s general condition worsened, being transferred to the intensive care unit, the evolution was unfavorable followed by death. The necropsy showed a left bronchopulmonary neoplasm with liver, kidney and adrenal metastases, a recent myocardial infarction (MI), a lung infarction and the histological result confirms the existence of small cell lung cancer (SCLC).

## Discussion

Patients with PE often have leg deep vein thrombosis (DVT), but sometimes, a DVT (peripheral or abdominal vein thrombosis) is not detected after accurate and sensitive testing.

Pathophysiology of cancer-associated thrombosis stems from the hypercoagulable state related to the increasing production of substances like: tissue factor (TF), podoplanin (PDPN), neutrophil extracellular traps (NETs), and plasminogen activator inhibitor-1 (PAI-1) that has high procoagulant activity [8]. 

TF is a glycoprotein receptor and cofactor for factors (F)VII and FVIIa. The TF/FVIIa complex is the physiological initiator of the extrinsic pathway of blood coagulation. TF is expressed by most types of tumors. Gene mutations are associated with increased TF expression in some types of cancer. For example KRAS, tumor protein 53 and phosphatase and tensin homolog gene mutations are associated with increased TF mRNA expression in tumors in patients with NSCLC. ALK rearrangement and STK11 gene mutation are also associated with VTE in patients with lung cancer [8]. 

PDPN is expressed in different types of cancer, including mesothelioma, seminoma, and glioma. Citrullinated histone H3 (H3Cit) and H3Cit-DNA complexes are the best biomarkers for NETs [9]. 

PAI-1 is a serine protease inhibitor that inhibits tissue plasminogen activator (tPA) and urokinase-type plasminogen activator (uPA) and thereby reduces the generation of plasmin. Increased levels of circulating PAI-1 are associated with thrombosis [8].

Malignancy is an important risk factor for PE.The presentation as PE without DVT instead of DVT/PE with DVT can be possible, because of risk factors and multiple, overlapping thrombotic and hemostatic pathophysiological pathways that are specific to these patients.

Given that VTE may be the first manifestation of cancer, there are many studies looking at different occult cancer screening strategies in this category of patients. Although the occurrence of cancer in patients who develop unprovoked VTE is not that high, recent research is geared towards identifying patients in this category who are at higher risk of occult cancer detection. For patients with a history of unprovoked VTE, age over 60, male gender and cigarette intake may constitute relevant indicators regarding the detection of occult cancer [10]. To identify cancer risk in patients with unprovoked VTE, a score based on data from the Registro Informatizado Enfermedad TromboEmbolica (RIETE score) has been developed for this category of patients. Components of this score include male sex, age over 70, anemia, chronic lung disease, thrombocytosis, and recent surgery. A score ≤ 2 was associated with an occult cancer risk of 5.8%, while a score ≥ 3 was associated with an occult cancer risk of 12% [11]. Even if clinical practitioners have not yet adopted the use of this score in recent guidelines, researchers have taken an interest in it. There have been several large studies answering the question of whether cancer screening should be performed in patients with unprovoked VTE and looking at limited and extensive screening strategy. In the multicenter SOME (Screening for Occult Malignancy in Patients with Idiopathic Venous Thromboembolism) clinical trial, 854 patients with unprovoked VTE were randomized to limited screening (medical history, physical examination, routine blood tests, chest X-ray) as well as to gender- and age-specific cancer screening (breast cancer, cervical, prostate, lung, colon) according to national guidelines, or extended screening, which included additional computed tomography (CT) of the abdomen, pelvis and chest, abdominal and pelvic ultrasound, endoscopy, mammography, serum tumor markers and more recently positron emission tomography/computed tomography (PET-CT). The study showed a cancer prevalence of 3.2% in the limited screening group and 4.5% in the more extensive screening [11, 12]. Another study, which included 195 patients with unprovoked VTE, compared the strategy of expanded CT screening in combination with fecal occult blood testing, showed no statistical benefit of it over limited screening [13]. Due to the failure of expanded screening to demonstrate its usefulness in terms of a higher rate of cancer diagnosis, detecting early-stage tumors, or improving patient outcomes, the International Society on Thrombosis and Hemostasis (ISTH) in 2017 and the National Institute for Health and Care Excellence (NICE) in 2012, and more recently 2020 guidelines came out with the recommendation that patients with unprovoked VTE undergo limited screening [14]. However, it remains unclear whether the subgroup of patients at increased risk of cancer should benefit from an expanded screening strategy from the outset. Although not yet approved, trying to relate the RIETE score to our case, the patient would have a score equal to 2, which would mean that when the diagnosis of pulmonary embolism (PE) was made the patient was at intermediate risk of cancer detection, which is why he should have been subjected to the screening strategy from the outset. Early diagnosis of bronchopulmonary cancer may have allowed appropriate therapeutic measures to be taken, which would have reduced the risk of mortality and increased the patient’s survival time.

When facing a patient with a first unprovoked episode of VTE, one must search for antiphospholipid syndrome (APS), as a cause of the venous thromboembolism. In our patient, the age, the absence of familial history for this condition, and the fact that the patient was already on OAC with a direct oral anticoagulant (DOAC), made us restrain from testing for thrombophilia during hospital stay. However, if there is a high clinical suspicion of APS, testing for APS associated antibodies should be considered. ELISA-based antibody tests for anti-cardiolipin antibody and beta-2 glycoprotein 1 antibody are not affected by anticoagulation. Testing for lupus anticoagulant (LA) in patients on OAC may have false positive results. It is recommended to switch to a LMWH if on OAC drug, and to perform testing for LA 12 h after the last administration of the parenteral drug [15, 16].

VTE may be the first sign of undiagnosed cancer and is not limited to PE and DVT, there are increasing reports of unusual thrombosis and arterial thromboembolism (ATE), including MI and stroke.

A recently published US study demonstrated increased risk of ATE 5 months before occult cancer diagnosis and progressive increase, with a fivefold increased risk in the last month before diagnosis [11]. This was most likely encountered in the evolution of our case, the patient having had a transient ischemic attack during hospitalization and had recent myocardial infarction changes at necropsy.

The absolute risk of VTE and ATE in patients with cancer varies widely depending on the site, stage, and time after diagnosis. Primary brain tumors, pancreatic, stomach, esophageal, ovarian, and lung cancers confer high risk of thromboembolism [17]. Regional or metastatic spread is associated with a higher risk of VTE compared to localized disease [17]. Approximately 50% of patients presenting with VTE at the time of diagnosis have synchronous metastasis. Timing is also important, patients are at the highest risk in the first 3 months after cancer diagnosis, followed by a declining incidence [18]. According to a large retrospective cohort study, the incidence of ATE at 6 months was higher in patients with lung, gastric, and pancreatic cancers (8.3%, 6.5%, and 5.9%, respectively) [19].

VTE is highly recurrent. Several studies have attempted to demonstrate recurrence rates of VTE associated with cancer, particularly occult cancer. One study including 733 patients with unprovoked VTE, showed that 110 cases had obvious cancer and 40 patients suffered from occult cancer. The  1-year cumulative incidence of VTE recurrence was 38.6% in subjects with occult cancer, and 15.5% in patients with obvious cancer. The 1-year risk of recurrence in subjects with occult cancer was 12-fold higher and 4-fold higher in cases with obvious cancer than in non-cancer patients. At the time of recurrent VTE and the occurrence of arterial thromboembolism, the patient is under OAC treatment (initially with rivaroxaban, changed to LMWH after thrombosis recurrence), according to the guideline recommendations [20].

VTE in cancer is not limited to DVT and PE, with increasing reports of unusual site thrombosis, including the upper extremities, cerebral veins, and splanchnic veins. Splanchnic or visceral vein thrombosis is frequently associated with cancer, especially certain gastrointestinal malignancies. Most of those findings are incidentally discovered on routine surveillance or restaging scans, and their potential impact on prognosis and outcomes is still uncertain. In contrast, ATE predominantly manifests as MI and cerebrovascular event, diseases that are hardly incidental because of their significant clinical impact [21]. 

One study, which analyzed patients having overcome an initial episode of acute PE after discontinuation of treatment, found the recurrence rate to be 4.5% per year, compared with 2.5% per year after PE occurring in the absence of a transient risk factor or known cancer [20].

The most common neoplasms associated with VTE recurrence were lung and gastrointestinal cancers, which are more prothrombotic and are detected in advanced stages. The majority (69%) of VTE recurrences in subjects with occult cancer took place before or shortly after they had been diagnosed with cancer [4]. Returning to our case, the sudden deterioration of the general condition with the onset of acute respiratory failure, subsequently resulting in the death of the patient, could be explained by a recurrent PE despite adequate anticoagulant treatment (Fig. 4). Unprovoked thromboembolic events may be the first sign of an occult cancer, although this is known and reported in a low incidence, thus being underestimated. The case presented above describes the likelihood of unprovoked venous and arterial thromboembolism in the same patient and highlights the impact on the survival rate of undiagnosed early pathology and how important the correct screening of patients with risk factors is.

Splenectomy, a standard procedure for many hematological conditions, is associated with an increased risk of late vascular complications. A publication conducted on the hereditary spherocytosis database at the University of Wisconsin Medical Center [22], which has had an interest in hereditary spherocytosis for half a century, reported that the rate of cardiovascular events after age 40 was five times higher in patients with hereditary spherocytosis without spleen than in patients with hereditary spherocytosis with spleen. Under these circumstances, VTE in our case could also be related to the history of hereditary spherocytosis, only the possibility of extensive evaluation could have elucidated this. Further research is needed to understand the pathophysiological mechanisms behind delayed thromboembolic complications in patients with hereditary spherocytosis and splenectomy.

## Conclusions

Unprovoked venous thromboembolism may be the first sign of occult cancer. The risk of missing an early-stage cancer diagnosis by not screening can have negative implications for patients as well as clinicians, so patients with unprovoked VTE and risk factors for cancer should undergo extensive screening, in our opinion.

Our case could represent an example of extending delayed adverse vascular events to patients with hereditary spherocytosis and splenectomy and supports the possible association between splenectomy and increased risk of thromboembolic events.

## Data Availability

The original contributions presented are included in the article/supplementary material, further inquiries can be directed to the corresponding authors.
